# Perceived class climate and school-aged children's life satisfaction: The role of the learning environment in classrooms

**DOI:** 10.1371/journal.pone.0189335

**Published:** 2018-02-08

**Authors:** Katharina Rathmann, Max G. Herke, Klaus Hurrelmann, Matthias Richter

**Affiliations:** 1 Institute of Medical Sociology, Medical Faculty, Martin Luther University Halle-Wittenberg, Halle (Saale), Sachsen-Anhalt, Germany; 2 Department for Sociology of Rehabilitation, Faculty of Rehabilitation Sciences, Technical University Dortmund, Dortmund, Germany; 3 Hertie School of Governance, Berlin, Germany; TNO, NETHERLANDS

## Abstract

The aim of this study is to examine the impact of class-level class climate on school-aged children’s life satisfaction. Data was derived from the German National Educational Panel Study (NEPS) using sixth grade school-aged children (n = 4,764, 483 classes). Class climate includes indicators of teachers' care and monitoring, demands, interaction, autonomy, as well as school-aged children's attitudes towards schoolwork at the class- and individual-level. Results showed that individual perceived class climate in terms of teachers' care and monitoring and autonomy was positively related to life satisfaction, whereas school-related demands were related to lower life satisfaction. Besides teachers' care and monitoring at class-level, indicators of class climate were not associated with school-aged children’s life satisfaction, while the individual perceived class climate is more important for life satisfaction.

## Introduction

The school is a key context for young people's development–ranging from the breadth and depth of their intellectual capital and nature of peer influence to their wellbeing [1, 2]–as students spend a long time of their daily life in school [3, 4]. Subjective wellbeing can include either cognitive judgments, such as life satisfaction, or emotional events, for example, feeling positive emotions [4, 5]. Life satisfaction as an evaluation of an individual’s quality of life, is an important aspect of wellbeing [5, 6] that is closely linked to subjective health [7], social competence and good coping skills [8]. Prior studies revealed that life satisfaction is not only an important predictor of life outcomes in adulthood, but it is also important in predicting the development of young people [9–12].

The majority of previous research focused on individual socio-demographic and non-school characteristics in order to explain differences in young people's life satisfaction [3, 10, 13]. However, only few have examined the importance of school-related features, such as class climate [1, 4, 14]. Although there is not yet a consensus about which dimensions are important for the valid measurement of class climate [15], the multidimensional concept of class climate generally refers to the social interaction between students and teachers in relation to collective beliefs, values and attitudes that prevail in classrooms [16–18]. Further, the class climate often refers to patterns of school-aged children's experiences of school and class life which reflects norms, values, interpersonal relationships, teaching and learning practices, as well as organizational structures [15]. Class climate has often been divided into different areas: (1) organizational structures and teaching and learning practices refer to, for instance, organization of teaching, such as teachers' monitoring in class and school-related demands, (2) interpersonal relationships in relation to schoolwork comprise interactions between teachers and students in class (i.e. teachers' allowance of interaction among classmates and student autonomy in class work); and (3) goals and values of classmates towards schoolwork (for example, expected effort, school-related ambition and disengagement from school), being regarded as relevant characteristics of school-aged children's perception of class climate [19–23].

Firstly, with regard to organizational structures and teaching and learning practices in class in particular, schools have many parallels with adult work settings, being characterized by deadlines, authority hierarchies and limited control over tasks and activities [24, 25]. According to the self-determination theory [26] evidence suggests that teachers who support students’ basic psychological needs for autonomy, competence, and relatedness facilitate school-aged children's autonomous self-regulation for learning, academic performance and wellbeing [27]. In relation to school-aged children's wellbeing, previous studies have shown that a higher teachers' monitoring in class and higher school-related demands in class are likely to be negatively associated with feelings of pressure and low wellbeing among students [17, 28–31].

Secondly, with regard to class climate indicators belonging to interpersonal relationships in relation to schoolwork often comprise interactions between teachers and students in class (i.e. teachers' permissiveness of interaction among classmates and autonomy in classwork). For instance, in relation to interaction and autonomy, it has been shown that interaction among classmates during lessons is related to better wellbeing among students, while a high level of school-aged children's autonomy over schoolwork in class tends to be positively linked to students’ wellbeing [32]. Further, better interpersonal relations and student-teacher relations were associated with higher life satisfaction–representing a measure of cognitive wellbeing [33–35].

Thirdly, also issues of class climate such as goals and values of classmates towards schoolwork (for example, expected effort, school-related ambition and disengagement from school) have been considered as relevant characteristics of school-aged children's perception of class climate [19–23] as adolescence is clearly characterized by a strong reliance on peers, classmates are important reference groups in classrooms [1]. In this context, goals and values of classmates towards schoolwork, such as their expected effort from other classmates, their school-related ambition and disengagement from school, shape the overall learning environment in class. In general, previous research work revealed that expectations towards schoolwork from others (for example, from teachers or peers) may make students feel pressured, which is likely to result in poorer wellbeing [30–32]. In contrast, ambition towards schoolwork relates to school-related aspirations and engagement in schoolwork. In this context, studies have highlighted that higher disengagement in schoolwork–which is linked to a less conducive learning and more disruptive behavior in class [1, 36, 37]–is negatively related to students’ wellbeing [38, 39].

Overall, prior studies have attempted to explain students’ life satisfaction mostly by those individual perceptions of class climate features, reported by students. However, from a theoretical point of view it is further likely that not only the individual perception of class climate features is important for young people’s life satisfaction, but also the social context, in which young people live and learn [1, 32]. At school-age, students are embedded in social contexts of schools and classrooms, characterized by a different intake and composition of students, and which are likely to shape the overall learning environment in schools and classrooms. In this context, it is likely that also the school and class context is important in contributing to young people's life satisfaction as they share a certain learning environment in schools and particularly in classes during school days [40, 41]. Thus, classrooms constitute the most important psychosocial environment of educational settings for young people in terms of the learning climate, cooperation, competition, student participation and school engagement, but also in terms of shared beliefs, emotions, habits and peer pressure, also having an impact on school-aged children’s wellbeing in both positive and negative ways [1].

From a theoretical point of view, particularly adolescence is a vulnerable stage in life, characterized by increased needs for autonomy as well as self- and relationship development with peers and classmates [1], but is also a period when comparisons with other peers increase and reference group mechanisms in relation to comparisons of social and scholastic issues, such as socioeconomic position or school performance, may be enforced. It is therefore likely that comparison processes in classrooms increase during adolescence which may cause some students to question their academic abilities, to feel that their self-esteem is being threatened and probably make them feel unhappy [42, 43]. According to the developmental mismatch theory, mismatches between these needs and the learning context are likely to contribute to poor adjustment and low wellbeing among young people [25, 44]. Many studies have shown that, for instance, low-achieving students placed in classes with on average higher academic performance, reported lower general and academic self-concept compared to high performing students [45, 46]; a finding which is clearly related to reference group mechanisms and social comparisons with classmates. Therefore, the composition of students in classrooms creates different learning environments [47, 48], while some classes enjoy a positive climate where students are supportive, devoted, and contribute to the functioning of the class; others are characterized by a negative climate with higher levels of peer pressure and comparison processes [49].

From a methodological point of view, the school environment has often been seen as a “multi-layered phenomenon” [47], being composed of individual students, classrooms and the broader school context. In recent years, studies have increasingly begun to methodologically consider this multilevel phenomenon in hierarchical multilevel models. Features of school-aged children's composition in class–well-known as so-called “compositional” characteristics–are operationalized by aggregating individual information from school-aged children to the class- or school-level in order to represent the overall mean-level or share of school-aged children in class [40, 47]. In terms of statistical modeling, this is usually achieved by averaging the same individual-level information on indicators of interest from individual students in class to the class- or school-level in order to represent the class average or mean-level of school-aged children in class and to segregate individual level indicators from those at higher levels (i.e. class- or school-level) [21, 49–51]. In general, this approach is required in order to examine and to better understand the impact of independent school- or class-level indicators on school-aged children’s outcomes as well as possible mechanisms leading to school class variations, for instance, in school-aged children's life satisfaction [4] or in other outcomes [45, 52, 53]. For instance, a study using data from students in grades 7 and 9 from Sweden revealed that a lower overall degree of peer acceptance in school classes is associated with poorer psychosomatic health complaints among female school-aged children [49]. Eriksson & Sellström [52] demonstrated a substantial variation between school classes in school-aged children’s ‘ subjective health complaints, by highlighting that in school classes with high demands, the odds of having subjective health complaints was about 50% higher than in school classes with low demands. Another study from Sweden showed that male school-aged children in grades 7 through 9, attending schools with an above median proportion of school-aged children who do not experience clear behavioral rules in school, decreased the odds ratio of poor self-reported health [54].

Regarding the perceived climate in school classes as a shared learning environment little is known about its impact on life satisfaction among young people. There are only few multi-level studies focusing on particularly school-aged children’s life satisfaction, so far, by taking individual indicators as well as the class composition in terms of the overall learning environment derived from the combination of these individual indicators into account. For instance, there is a study from Western Canada which revealed that an increase in overall level of school connectedness at the school-level was associated with higher life satisfaction [4]. However, many multilevel studies, taking class or school compositional characteristics into account, did not focus on life satisfaction, rather than on other outcomes, such as self-rated health, psychosomatic health complaints and emotional symptoms, or used various indicators of school or class climate [45, 53, 55]. Thus, it is difficult to draw general conclusions of the state-of-the-art as previous research used heterogeneous outcomes of wellbeing–either multidimensional or one-dimensional items of wellbeing, such as life satisfaction–or focused only on single measures of school or class climate. Thus, studies using features of the overall learning environment in class in relation to school-aged children’s life satisfaction, by taking the multilevel structure of schools into account are further warranted. So far, not much is known about whether the overall learning environment in terms of class climate in classes is likely to have an independent contribution to school-aged children’s life satisfaction [40, 53].

In sum, studies using multilevel analyses to disentangle and quantify the importance of the individual perception of the learning environment and the overall learning environment in classrooms for school-aged children’s life satisfaction are few and findings often vary from study to study due to the use of different school or class climate indicators or outcomes of wellbeing [55]. Yet, these relationships are important to investigate because they suggest different implications for school policies in order to ameliorate school-aged children’s overall wellbeing. Due to the lack of research on the role of the overall learning environment in classrooms on life satisfaction, the purpose of our study is to investigate whether the overall learning environment in terms of class climate is related to school-aged children's life satisfaction above and beyond school-aged children's individual perception of the class climate.

In this study we hypothesize that the overall learning environment in terms of class climate in classrooms is associated with young people's life satisfaction over and above school-aged children's individual perception of the class climate. According to the self-determination theory [26], school-aged children should be curious about the school environment and should be interested in schoolwork and learning. However, teachers often introduce external controls into learning climates, which can undermine the sense of relatedness between teachers and school-aged children [27], which is contradicting to school-aged children's will of self-determination. Thus, teachers with an autonomy-supportive teaching style and who allow interaction among school-aged children during schoolwork in class, create a conducive learning environment among peers in class, which provide opportunities for the school-aged children to feel autonomous, competent, and emotionally supported [1]. In line with the developmental mismatch theory [1, 44], we assume that school-aged children being placed in a classroom with an overall higher level of teachers' care and monitoring is negatively associated with school-aged children’s life satisfaction as they may feel under teacher supervision and are thus likely to feel pressured regarding schoolwork in class. In contrast, a higher extent of autonomy and interaction among school-aged children in classrooms will be positively related to young people's life satisfaction. Further, given the above elaborated centrality of peers and classmates in adolescence, also social comparison processes with the reference group as well as expectations by classmates towards schoolwork and academic effort are likely to be related to school-aged children’ life satisfaction. Classmates who require effort from other peers are likely to put pressure on those school-aged children, which challenges their psychological needs for competence and relatedness, being detrimental for their overall life satisfaction. In this context, it is likely that school-aged children being surrounded by peers with an overall higher level of ambition or expecting an overall higher level of academic effort in classes may be detrimental for life satisfaction as the learning climate is characterized by comparisons with the reference group and peer pressure in terms of competition. However, also the contrary may be hypothesized in relation to the class-level extent of classmates who are ambitious towards schoolwork because school-aged children's school-related ambition is generally related to higher motivation and school engagement which could also positively affect classmates' life satisfaction. Finally, a higher level of school-related disengagement in classrooms is likely to be associated with poorer life satisfaction among school-aged children as the overall atmosphere in class may be characterized by a more negative and detrimental climate that is less conducive and productive for the overall learning situation in class.

## Materials and methods

### Data and sample

The National Educational Panel Study (NEPS), carried out by the Leibniz Institute for Educational Trajectories (LIfBi) at the University of Bamberg, analyzes educational processes in Germany from early childhood to late adulthood. Besides comprehensive competence tests covering several domains (language, mathematics, sciences), NEPS also surveys non-cognitive measures, such as subjective health and life satisfaction [56].

NEPS representatively selects and surveys school-aged children who attend regular or special schools and are willing to participate to be questioned and tested annually [56]. The NEPS data collection is part of the “Framework Program for the Promotion of Empirical Educational Research” funded by the German Ministry of Education and Research and supported by the federal states. For school-aged children of the starting cohort 3 (SC3, fifth graders), the first survey (wave 1) was carried out in the classroom via Paper and Pencil Interviews (PAPI) in fall/winter 2010 and the second survey in winter 2011/12. Students of the cohort were sampled through a stratified multi-stage process. First schools were sampled after stratification by school type, region and other characteristics. In the next step, classes were sampled and school-aged children from those classes comprised the final sample [57, 58]. The survey documents used were submitted, reviewed and approved by their respective Ministries of Education of the 16 federal states. Federal states were responsible for the compliance with the statutory data protection regulations of the NEPS [59]. Only those school-aged children were included in the survey who obtained consent from parent or guardian. In addition to the surveying and testing of school-aged children, parents, teachers, and school principals are also part of the NEPS surveys, where applicable [56].

In this study, we focus on individual measures from school-aged children, who were surveyed in survey 2011/2012 of the NEPS SC3, as this survey–in comparison to the first survey–includes a variety of class climate indicators. A total of 5,525 school-aged children from regular schools participated in this survey 2011/2012, including those in regular schools in lower secondary education or those who were still in elementary school, as some federal states in Germany extended the elementary schooling until grade 6 (for example, Berlin and Brandenburg) before tracking into different school types takes place. Schools for school-aged children with special educational needs were excluded from the analyses due to differences in questionnaires limited comparability. Mean age in grade 6 was 11.5 years (SD: 0.6) and 51.6% of the sample were boys. In total, 68.5% of school-aged children report to live with both parents, 12.7% live in single parent families, 11.0% in step families, and 5.7% in another family structure, such as with foster-parents. The German school system is organized very hierarchically with a “tripartite” structure of school types, being characterized by the co-existence of different tracks in secondary education with different requirements and learning environments, and a high social segregation (i.e. by parental socioeconomic background) among different school types. The majority of school-aged children attend high track schools (i.e. “Gymnasium”, 43.7%), whereas 21.3% attend medium track (i.e. “Realschule”), 21.5% attend comprehensive schools (i.e. “Gesamtschule”) and 13.5% attend low track schools (i.e. “Hauptschule”).

### Instruments

#### Dependent variable

General life satisfaction was used as an indicator of school-aged children's cognitive wellbeing. Life satisfaction was measured by asking school-aged children how satisfied they are with their life (0 =“not at all”– 10 = “very satisfied”) [60]. Life satisfaction has been validated in several studies [61] and was used as a metric measure in our analyses.

#### Independent variables

In order to examine the role of the overall learning environment in classrooms, the NEPS data provides relevant indicators of the class climate in the survey 2011/2012 of starting cohort 3, which are measured at the individual level, asking school-aged children about several issues in relation to the learning situation in their school classes. Four standardized constructs of school-aged children's individual perception of class climate features (i.e. teachers' care and monitoring, school-related demands, autonomy and interaction among school-aged children in class) were included as sum scores with moderate to high internal consistencies (Cronbachs alphas: 0.67–0.84), respectively (see Table 1 for description of items and sum score indices). Further, three single-items have been used in relation to school-aged children's individual attitudes toward school-related goals, values and perception of schoolwork (Table 1) which reflect the individual school-aged children’s perception of the general learning environment. These seven variables provide the individual-level indicators for perceived class climate.

**Table 1:** Indicators of perceived class climate.

Variables	Item(s)	Operationalization	Cronbach‘s Alpha	Source
Teacher care and monitoring *	I think my German teacher:	1 = does not apply… 5 = applies completely (Range: 4–20)	~ 0.82	[62] (Item 1), [63] (Items 2–4)
	is aware of everything that happens in class.			
	manages to quickly involve me again, if I don't pay attention for a moment.			
	instantly notices when I don't pay attention.			
	has the class under control.			
Demands *	I think my German teacher:	1 = does not apply … 5 = applies completely (Range: 5–25)	~ 0.67	[62] (Items 1–3), [36] (Items 4–5)
	expects me to try my very best.			
	tells me that she/ he thinks that I can do better than I have done so far.			
	finds it very important that we do our work very thoroughly.			
	uses students that achieve good grades as an example for us all.			
	tells us where we stand compared to our classmates.			
Autonomy *	I think my German teacher:	1 = does not apply… 5 = applies completely (Range: 3–15)	~ 0.82	[64]
	first tries to understand my point of view, and then tells me what she/he would do.			
	listens to my suggestions and takes them seriously.			
	encourages me to ask questions.			
Interaction *	I think my German teacher:	1 = does not apply… 5 = applies completely (Range: 3–15)	~ 0.84	[36]
	allows us to discuss our assignments with each other			
	encourages us to help each other in class.			
	encourages us to exchange ideas with each other in class.			
Ambition of classmates	Most of my classmates are very ambitious at school.	1 = does not apply… 5 = applies completely (Range: 1–5)	-	NEPS
Expected effort by classmates	Most classmates expect classmates to make an effort at school.	1 = does not apply… 5 = applies completely (Range: 1–5)	-	NEPS
School disengagement of classmates	Most classmates don't care how well classmates do at school.	1 = does not apply… 5 = applies completely (Range: 1–5)	-	NEPS

Note

* these indicators have been used as index variables, based on validated constructs and with moderate to high internal consistency.

Further, for each of the seven individual-level indicators of perceived class climate, an aggregated mean score of the same indicators was created at the class-level for each class and was centered on its grand mean [50, 51]. Table 2 presents a description of all measures, corresponding items and internal consistencies of index variables, respectively, that have been created. These seven aggregated variables provide the class-level indicators and present the overall class climate at class-level.

**Table 2:** Sample description (NEPS SC3, survey wave 2011/2012, N = 5,525).

			Frequencies	
			absolute (n)	relative (%)
**Gender**				
Boy			2,852	51.6
Girl			2,666	48.3
Missing			7	0.1
**School type**				
High track („Gymnasium“)			2,415	43.7
Medium Track („Realschule“)			1,175	21.3
Low track („Hauptschule“)			745	13.5
Mixed track (comprehensive school type and elementary schools)			1,190	21.5
Missing			0	0
**Family structure**				
both parents			3,787	68.5
single parent			703	12.7
Step parents			552	10.0
Other family structure			317	5.7
Missing			166	3.0
	**Cronbach‘s alpha**	**Missing % (N)**	**Mean (SD)**	**Min.-Max**.
**Wellbeing indicator**				
Life satisfaction	-	10.8 (598)	7.51 (2.27)	0–10
**Perceived class climate indicators**				
Teacher care and monitoring (4 Items)	0.82	14.4 (800)	13.75 (3.68)	4–20
Demands (5 Items)	0.67	16.6 (919)	16.85 (3.90)	5–25
Autonomy (3 Items)	0.82	16.7 (922)	10.21 (2.96)	3–15
Interaction (3 Items)	0.84	15.9 (880)	10.03 (2.95)	3–15
Ambition of classmates (1 item)	-	11.1 (614)	2.83 (0.95)	1–5
Expected effort by classmates (1 item)	-	11.2 (623)	2.37 (1.12)	1–5
School disengagement of classmates (1 item)	-	11.4 (630)	2.94 (1.19)	1–5

Note: SD = Standard Deviation

In research on compositional analyses it is a necessary precondition to include not only the class-level aggregates but also to adjust for the individual measures [47, 50, 65]. Therefore, all class climate indicators were also considered as measures at the individual level. All indicators have been z-standardized in order to compare coefficients of those indicators in relation to their impact on school-aged children's life satisfaction. Table 2 shows the distribution of indicators of class climate and life satisfaction.

We controlled for school-aged children's gender and family structure (families with both parents was used as reference category, single parent families, step families, and other types of families) in the analyses. Further, we used information on the attended school type [66] in order to control for possible socioeconomic differences among school-aged children in German lower secondary schools. As the educational system in Germany is highly differentiated and hierarchically organized in terms of different low (“Hauptschule”), medium (“Realschule”), high track schools (“Gymnasium”) and comprehensive schools (“Gesamtschule”) combining aspects of all tracks and comprising those school-aged children who attend the extended elementary schools until grade 6 we therefore control for this categorical variable in the analyses.

### Statistical analyses

The study utilizes multilevel analysis that allows the modelling of hierarchical or nested data structures [50, 51]. The level 1-units in the sample are individual school-aged children; the level 2-units are classes and level 3-units are schools. Multilevel analysis is based on the assumption that the regression constant (intercept) may vary for units on every level, i.e. individual-, class- and school-level, respectively, and may be explained by measures at those levels. The individual- and class-level determinants were included in the models using a stepwise approach. First, an empty model (Model 0) tested the Intraclass Correlation Coefficients (ICC), which represent the proportion of variance on latent school and class effects by indicating the variance in the outcome attributed to differences between schools and classes [50, 51]. The variance of the school- and class-mean of the outcome life satisfaction was examined in random intercept model with no additional indicators [51] (see Eq 1). Only manifest indicators at individual as well as their manifest aggregation at class level were used, thus considering the “taxonomy of contextual models” (compare [67]) this study uses a doubly manifest design.

$$\begin{array}{l} \mathrm{Level}\;1\;\left(\mathrm{individual}-\mathrm{level}\;i\right):\gamma_{ics}=\beta_{0cs}+E_{ics}\\ \mathrm{Level}\;2\;\left(\mathrm{class}-\mathrm{level}\;c\right):\beta_{0cs}=\gamma_{00s}+U_{0cs}\\ \mathrm{Level}\;3\;\left(\mathrm{school}-\mathrm{level}\;s\right):\gamma_{00s}=\sigma_{000}+V_{00s} \end{array}$$ (1)

The life satisfaction score of the individual school-aged child i in class c in school s (y_ics_) is a function of the mean life satisfaction score for class c in school s (b_0cs_) plus a residual term, reflecting an individual deviation from the predicted outcome (E_ics_). The mean life satisfaction score for class c in school s (β_0cs_) is a function of the school mean of life satisfaction in the sample (ɣ_00s_) plus another residual term, this time reflecting the class-specific deviation from the predicted outcome (U_0cs_). Finally, the school mean (ɣ_00s_) is a function of the grand mean (σ_000_) and school-specific deviation from the predicted outcome (V_00s_). Analysis of the unconditional model including both class-level and school-level suggested low but statistically significant variation in life satisfaction between school classes (ICC for classes: .017 = 1.7%), but very low and not significant variance in life satisfaction between schools (ICC for schools: .008 = .08%). Therefore, for Model 0, we decided to only conduct 2-level hierarchical models (school-aged children nested in classes). Model 1 considered gender, family structure and school type, as well as the indicators for perceived class climate at the individual-level (see Eq 2).

$$\begin{array}{l} \mathrm{Level}\;1\;\left(\mathrm{individual}-\mathrm{level}\;i\right):\gamma_{ic}\\ \;=\beta_{1c}\left(TeacherCare\;and\;Monitoring_{ic}\right)+\beta_{2c}\left(Demands_{ic}\right)\\ \;+\beta_{3c}\left(Autonomy_{ic}\right)+\beta_{4c}\left(Interaction_{ic}\right)+\beta_{5c}\left(Ambition_{ic}\right)\\ \;+\beta_{6c}\left(Effort_{ic}\right)+\beta_{7c}\left(Disengagement_{ic}\right)+\beta_{8}\left(Gender_{i0}\right)\\ \;+\beta_{9}\left(FamilyStructure_{i0}\right)+\beta_{10}\left(SchoolType_{i0}\right)+E_{1c}\\ \mathrm{Level}\;2\;\left(\mathrm{class}-\mathrm{level}\;c\right):\beta_{0c}=\gamma_{00}+U_{0c}\\ \beta_{1c}=\gamma_{10};\;\beta_{2c}=\gamma_{20};\;\beta_{3c}=\gamma_{30};\;\beta_{4c}=\gamma_{40};\;\beta_{5c}=\gamma_{50};\;\beta_{6c}=\gamma_{60};\;\beta_{7c}=\gamma_{70};\;\beta_{8}=\gamma_{80};\;\beta_{9}\\ \;=\gamma_{90};\;\beta_{10}=\gamma_{100} \end{array}$$ (2)

Model 2 additionally included all indicators of overall class climate at class-level (see Eq 3) that might explain this variation in life satisfaction. These are the class averages of the individual-level indicators of perceived class climate.

$$\begin{array}{l} \mathrm{At}\;\mathrm{level}\;1\;\left(\mathrm{individual}-\mathrm{level}\;i\right):\gamma_{ic}\\ \;=\beta_{1c}\left(TeacherCare\;and\;Monitoring_{ic}\right)+\beta_{2c}\left(Demands_{ic}\right)\\ \;+\beta_{3c}\left(Autonomy_{ic}\right)+\beta_{4c}\left(Interaction_{ic}\right)+\beta_{5c}\left(Ambition_{ic}\right)\\ \;+\beta_{6c}\left(Effort_{ic}\right)+\beta_{7c}\left(Disengagement_{ic}\right)+\beta_{8}\left(Gender_{i0}\right)\\ \;+\beta_{9}\left(FamilyStructure_{i0}\right)+\beta_{10}\left(SchoolType_{i0}\right)+E_{1c}\\ \mathrm{At}\;\mathrm{level}\;2\;\left(\mathrm{class}-\mathrm{level}\;c\right):\beta_{0c}\\ \;=\gamma_{00}+\gamma_{01}\left(AvgTeacherCare\;and\;Monitoring_{ic}\right)+\gamma_{02}\left(AvgDemands_{ic}\right)\\ \;+\gamma_{03}\left(AvgAutonomy_{ic}\right)+\gamma_{04}\left(AvgInteraction_{ic}\right)+\gamma_{05}\left(AvgAmbition_{ic}\right)\\ \;+\gamma_{06}\left(AvgEffort_{ic}\right)+\gamma_{07}\left(AvgDisengagement_{ic}\right)+U_{1c}\\ \beta_{1c}=\gamma_{10};\;\beta_{2c}=\gamma_{20};\;\beta_{3c}=\gamma_{30};\;\beta_{4c}=\gamma_{40};\;\beta_{5c}=\gamma_{50};\;\beta_{6c}=\gamma_{60};\;\beta_{7c}=\gamma_{70};\;\beta_{8}=\gamma_{80};\;\beta_{9}\\ \;=\gamma_{90};\;\beta_{10}=\gamma_{100} \end{array}$$ (3)

Furthermore, item-nonresponse in the raw data is evident and an analysis of complete cases would have led to a loss of 1,604 cases (29%) of the original sample. A comparison of cases with complete to those with incomplete information led to the conclusion, that the missing information might very well not be Missing Completely At Random (MCAR), but at least Missing At Random (MAR), leading to possibly biased estimates in analyses using listwise deletion [68]. For example, missing data is higher in male school-aged children (χ^2^ = 4.891, df = 1, p = 0.027), low track schools (χ^2^ = 236.237, df = 3, p<0.001), and low life satisfaction (χ^2^ = 32.532, df = 10, p<0.001). Therefore, multiple imputations were employed to minimise bias. All variables in model 1 (see Eq 2) were used in the imputation model, except for scales, of which the raw items were included instead. Dichotomous and categorical variables were imputed using logistic methods, predictive mean matching (PMM) was used to impute ordinal or metric variables. As preliminary analyses showed a negligible variance at school-level, only the class indicator was used for as an indicator for clustering. The dependent variable life satisfaction was included in the imputation model, but its imputed values not used for inference. Scales and class-means were computed after imputation. Missing information in life satisfaction and in the class indicator led to an effective sample size of 4,764 cases for the analyses. We imputed 25 data sets, balancing appropriate statistical power [69] with computational expenditure of time. The statistical analyses were conducted with the packages “mice” (“Multiple Imputation by Chained Equations”, [70] for imputations and “lme4” [71] for multilevel, the free software R version 3.3.2 [72].

## Results

### Descriptive results

Table 3 shows the pairwise correlations between the indicators of perceived class climate at the individual-level, respectively and school-aged children's life satisfaction. Indicators of individually perceived class climate are only weakly or not significantly correlated with young people's life satisfaction. Teacher care and monitoring is positively correlated with autonomy of school-aged children and interaction among school-aged children in classroom, indicating that higher levels of teachers' care and monitoring are related to higher perception of school-aged children’s autonomy and interaction. Further, school-aged children's perception of autonomy was also positively correlated with interaction among classmates.

**Table 3:** Pairwise correlations between class climate indicators at the individual-level (n_students_ = 4,764).

	Life satisfaction	Teacher care and monitoring in class	Demands	Autonomy	Interaction in class	Ambition by classmates	Expected effort by classmates	School disengagement
Life satisfaction	1							
Teacher care and monitoring	0.09***	1						
Demands	-0.01	0.36***	1					
Autonomy	0.11***	0.49***	0.35***	1				
Interaction in class	0.08***	0.41***	0.27***	0.60***	1			
Ambition of classmates	0.05***	0.09***	0.08***	0.11***	0.09***	1		
Expected effort by classmates	0.02	0.08***	0.15***	0.12***	0.11***	0.23***	1	
School disengagement of classmates	-0.08***	-0.03*	0.05***	-0.02	-0.05**	-0.03*	-0.08***	1

Note

Significance level * p < .05

** p < .01

*** p < .001

in bold: very high correlations (Pearson r > 0.4)

At the class-level (Table 4), indicators of the overall learning environment in classrooms are very weakly correlated with life satisfaction. The overall mean-level of teachers' care and monitoring in classroom is positively correlated with class-level autonomy and interaction, respectively, as well as with demands. Class-level autonomy is significantly correlated with higher levels of interaction between peers in classroom.

**Table 4:** Pairwise correlations between class climate indicators at the class-level (n_class_ = 483).

	Life satisfaction	Teacher care and monitoring in class	Demands	Autonomy	Interaction in class	Ambition by classmates	Expected effort by classmates	School disengagement
Life satisfaction	1							
Teacher care and monitoring	0.01	1						
Demands	-0.06***	0.50***	1					
Autonomy	0.03*	0.63***	0.41***	1				
Interaction in class	0.03*	0.57***	0.30***	0.79***	1			
Ambition of classmates	0.04**	0.12***	-0.03*	0.12***	0.11***	1		
Expected effort by classmates	0.00	0.11***	0.18***	0.14***	0.10***	0.29***	1	
School disengagement of classmates	-0.09***	-0.05***	0.15***	-0.05***	-0.06**	-0.10*	-0.11***	1

Note

Significance level * p < .05

** p < .01

*** p < .001

in bold: very high correlations (Pearson r > 0.4)

### Multilevel results

Table 5 shows the results from the hierarchical linear regression models. From the empty model (Model 0) it is evident that the largest variation in life satisfaction is due to individual differences between school-aged children. A statistically significant variation in life satisfaction between classes and schools does nevertheless exist. 4% of the variation in school-aged children’s life satisfaction can be ascribed to differences between the 483 classes, while the rest of the variation is due to individual characteristics (model 0).

**Table 5:** Parameter estimates for life satisfaction as a function of class climate at the individual- and class-level (NEPS SC3).

	Model 0	Model 1	Model 2
Individual variables	b-coefficients (SE)	b-coefficients (SE)	b-coefficients (SE)
Gender (Ref.: boys)			
Girls		-0.112 (0.065)	-0.118 (0.065)
Family structure (Ref.: both parents)			
Single parents		-0.526 (0.101)***	-0.510 (0.101)***
Step families		-0.505 (0.110)***	-0.503 (0.110)***
Other family structure		-0.586 (0.146)***	-0.574 (0.146)***
School type (Ref.: high track)			
Low track		-0.703 (0.120)***	-0.646 (0.133)***
Medium track		-0.265 (0.097)**	-0.251 (0.104)*
Comprehensive school		-0.232 (0.095)*	-0.186 (0.101)
**Class climate at the individual-level**			
Teacher care and monitoring		0.130 (0.039)***	0.184 (0.045)***
Demands		-0.095 (0.038)*	-0.091 (0.041)*
Autonomy		0.213 (0.047)***	0.206 (0.049)***
Interaction in classroom		0.009 (0.044)	0.022 (0.048)
Ambition of classmates		0.062 (0.036)	0.066 (0.038)
Expected effort by classmates		-0.016 (0.030)	-0.007 (0.032)
School disengagement of classmates		-0.119 (0.028)***	-0.101 (0.029)***
**Class climate at the class-level**			
Teacher care and monitoring			-0.213 (0.101)*
Demands			-0.005 (0.109)
Autonomy			0.046 (0.153)
Interaction in classroom			-0.032 (0.128)
Ambition of classmates			-0.040 0.124)
Expected effort by classmates			-0.112 (0.111)
School disengagement of classmates			-0.160 (0.091)
Intercept	7.489 (<0.001)***	8.106 (0.157)***	8.852 (0.453)***
Variance between classes	0.206 (0.454)	0.095 (0.309)	0.086 (0.293)
ICC_classes_	4.0%	1.2%	1.0%
N_classes_	483	483	483
N_students_	4,764	4,764	4,764
Deviance (-2x LL)	21,287.41 (df = 3)	21,118.74 (df = 17)	21,112.46 (df = 24)

Note: Ref. = reference category

Significance level * p < .05

** p < .01

*** p < .001

SE = standard errors; LL = Log Likelihood; df = degrees of freedom. Model 0: Empty model (without covariates), Model 1: individual-level variables of class climate; Modell 2: full model.

From model 1 is evident that school-aged children living in single parent, step- or other types of families report lower life satisfaction compared to male school-aged children or school-aged children living with both parents. Further, school-aged children attending medium and low track schools as well as comprehensive schools show lower life satisfaction in contrast to high track school-aged children. Life satisfaction does not significantly differ among gender.

Findings in model 1 show that school-aged children’s individual perception of the class climate is in part significantly related to school-aged children’s life satisfaction. Students, who perceive higher teachers' care and monitoring and autonomy show higher life satisfaction. In contrast, perceived demands as well as expected effort for school work by classmates were associated with significant lower life satisfaction among school-aged children. Perceiving classmates as disengaged from schoolwork is significantly associated with lower life satisfaction. Comparing the coefficients of all class climate indicators at the individual-level, the perceived autonomy showed the strongest impact on school-aged children life satisfaction compared to the other class climate indicators. These associations remained stable and significant in the final model including all individual and class-level indicators, simultaneously.

With regard to the overall learning environment in school classes, the indicators of the overall level of the learning environment in classes are included in model 2, controlling for gender, family structure and school type as well as school-aged children individual perception of class climate. On average, school-aged children being placed in a classroom with higher teachers' care and monitoring is negatively related to life satisfaction (model 2), indicating that an increase in average teachers' care and monitoring in classrooms was associated with a decrease in school-aged children life satisfaction. This result indicates that above the individual perception of class climate, also the average level of teachers' care and monitoring in class is associated with lower life satisfaction among school-aged children. Further, school-aged children being surrounded by classmates, who are perceived as being disengaged from schoolwork, report lower life satisfaction. This finding suggests that a learning environment in class that is perceived as less conducive to learning seems to be negatively associated with school-aged children’s life satisfaction. The other characteristics of the overall learning environment at the class-level did not show significant associations with life satisfaction in our sample.

Last, the deviances of the null model (model 0: -2x Log Likelihood = 21,285.17, df = 4), the intermediate model (model 1: -2x Log Likelihood = 21,118.74, df = 17), and the full model (model 2: -2x Log Likelihood = 21,112.46, df = 24), allow for an estimation of model fit. Model 1 is significantly better than model 0 (χ^2^ 221.49, df = 13, p<0.001). Model 2 fits slightly but significantly better than model 1 (χ^2^ 14.82, df = 7, p = 0.04), indicating that the inclusion of class climate as compositional measures at the class-level proves relevant. Thus, the final model revealed the best model fit.

## Discussion

### Summary of results

Our study is among the first analyzing the role of school-aged children’s individual perception of the class climate and the overall level of the learning environment on school-aged children's life satisfaction in Germany, taking into account individual student-level and aggregated class-level characteristics of class climate. The results showed that almost all of the variability in life satisfaction between schools and classes was explained by school-aged children’s individual perception of class climate. In line with previous studies, our findings highlighted that individual perceptions explained most of the variation in life satisfaction, while the importance of the overall learning environment in school classes were only to a small degree important for young people's life satisfaction [17, 54, 55, 73].

With regard to school-aged children’s individual perception of class climate, school-aged children who reported to perceive higher teachers' care and monitoring and higher autonomy showed higher life satisfaction, whereas adolescents who reported higher school-related demands and who perceive their classmates as disengaged from schoolwork revealed lower life satisfaction. Although the major part of the variation in life satisfaction operated at the individual-level, our findings indicated that above the individual perception of the class climate, the overall learning environment in school classes was partially related to school-aged children’s life satisfaction. School-aged children, being placed in classrooms with a higher level of teachers' care and monitoring, reported lower life satisfaction. However, other class-level characteristics of the learning environment (for example overall level of demands, autonomy, and interaction among school-aged children in classroom as well as classmates' school-related attitudes towards schoolwork) were not related to school-aged children's life satisfaction.

### Interpretation of results

With regard to the overall learning climate in school classes–measured as the mean-level of school-aged children reports on class climate indicators at the class-level–the results showed only a very little association of class-compositional measures with school-aged children's life satisfaction over and above those found at the individual-level. Our findings showed that school-aged children placed in classes where they were surrounded by classmates who perceive teachers on an above-average level caring and monitoring revealed lower life satisfaction. Thus, school-aged children in classrooms with higher levels of teachers who care and monitor school-aged children in class may feel observed by their teacher which limits their feeling of autonomy and self-determination [1, 44]. Our results for the class-level indicators of the overall learning environment in class are partially in line with findings from other studies using emotional symptoms or self-rated health as outcomes [17, 54]. Another study revealed that a higher share of school-aged children in class who reported that their opinions are taken seriously, that their teachers give praise, that most of their teachers provide interesting teaching, and that they can get immediate help with their schoolwork if needed, had significantly better self-rated health [17]. Students in our sample are in a vulnerable developmental phase, when young people increasingly rely on their classmates and peer groups. Thus, it is likely that high levels of care and monitoring by teachers reduces school-aged children's feeling of autonomy and life satisfaction. Poor psychological and behavioral adjustment has often been related to stressors [74, 75] and school can be seen as a source of distress amongst adolescents [25]. The school-aged children’s notion of autonomy and monitoring is formed by the regulations and framework for activities in school and the responsibilities that the school-aged children are given. The fairness and relevance of these regulations, and the extent to which school-aged children are allowed to influence their working conditions, are likely to influence the way in which school-aged children adjust to the school environment, and consequently, how they feel about school [76]. Participatory and autonomous learning processes may leave school-aged children with the experience that schooling has an intrinsic interest to them and thus has a positive influence on school adjustment and wellbeing [77]. Therefore, it is likely that the school-aged children in this study showed lower life satisfaction when they were placed in classrooms with a higher level of teachers' care and monitoring.

Surprisingly, in contrast to the overall level of teachers' care and monitoring in classrooms, we further revealed a positive association between school-aged children's individually perceived teachers' care and monitoring and life satisfaction that was not in line with our preliminary assumption and according to the developmental mismatch theory as our results showed higher life satisfaction among school-aged children who perceived higher care and monitoring by teachers. This finding may be explained by school-aged children's feeling of obtaining attention from and being cared as well as supported by their teacher. In this context, previous studies have also shown that support and care by teachers was related to better wellbeing [32]. Thus, school-aged children who perceive teachers as being very attentive, aware of everything that happens in class, manage to quickly involve school-aged children in class, are likely to feel better in terms of wellbeing.

Further, individually perceived autonomy was positively related to school-aged children’s life satisfaction, indicating that perceiving higher support and acknowledgment by teachers as well as being allowed to autonomously interact with other classmates during class, seems to nurture young people's need for self-determination, competence and relatedness with others, which is beneficial for school-aged children's life satisfaction. In contrast, school-related demands and expected effort by classmates was negatively associated with life satisfaction. Demands by teachers and expected effort by classmates are likely to be linked to feelings of pressure, such as behaving conforming to those demands and expectations, which hamper school-aged children overall life satisfaction.

Regarding the other characteristics of the overall learning environment in classrooms, such as the level of demands, autonomy, interaction, or academic ambition, expected effort of classmates and disengagement were not significantly related to school-aged children's life satisfaction. Thus, our hypotheses cannot be confirmed in relation to these indicators. According to our report of the state-of-the-art, it is very difficult to compare our results with those of previous evidence as they used either different outcomes of wellbeing or considered different measures of the overall learning environment in classes. For instance, a study from Sweden with school-aged children in grade 9 revealed that learning conditions in classrooms, such as school-aged children’s interaction and autonomy in class were also not related to school-aged children’s wellbeing, measured by psychosomatic health complaints [17]. Their study highlighted that particularly the overall level of teachers' help was related to school-aged children’s health complaints, indicating that the level of teacher support is important for young people's wellbeing in contrast to the level of autonomy or interaction in classrooms.

In relation to the other measures of the overall learning environment, such as the level of school-aged children in class who are disengaged from schoolwork, this association has not been examined regarding school-aged children’s life satisfaction. Other studies used, for instance, other measures such as the level of school connectedness at the school-level and showed that a higher share of school-aged children in school who reported low connectedness to school–as a kind of proxy measure for school disengagement–was associated with a higher risk of mental health problems among young people at school year 8 [78]. In general, school-aged children are likely to feel disconnected to and disengaged towards schoolwork if they are placed in a school or class that does not meet their developmental needs [1, 44], whereas feeling connected to and engaged towards schoolwork is increased when the social environment meets young people's developmental needs. However, further research is required that validate our findings because it might also be the case that the level of classmates who are perceived as being disengaged from schoolwork is likely to reflect the overall learning climate in class and relationship quality among classmates, which may be shaped by less cooperation among teachers and school-aged children in class as well as a more disruptive and negative learning climate. Lastly, it could also be plausible that the impact of the overall learning environment in classrooms is indirectly related to school-aged children's life satisfaction, operating through their association with resources or organizational and structural features of schools [47]. Thus, future studies should unravel these associations in further detail.

### Strengths and limitations

Strength of our study is the fact that it is based on a total sample, with school-aged children nested within classes and classes nested within schools, although the school-level was discarded from the final analyses due to low variation in life satisfaction among schools. This data structure, which is quite uncommon in these types of studies, makes it possible to carry out analyses at different levels of the school structure and to take measures at the class- and school-level into account. Due to low variation in life satisfaction between schools, we only considered the individual- and class-level, and thus, applied a two-level multilevel design.

However, there are some limitations that should be addressed. Both predictor and outcome variables rely on self-reported data, which raises the issue of negative affectivity [79]. Further, our study used a cross-sectional design in order to examine the impact of compositional class climate in classrooms over and above the individual assessed class climate on school-aged children’s life satisfaction. Thus, we are unable to make causal inferences. It is therefore likely that a negative class climate in classrooms affects schoolchildren’s life satisfaction, but school-aged children with low life satisfaction may also impact the class climate in classrooms. For instance, a comprehensive literature review [6] also showed that adolescents with higher levels of life satisfaction displayed higher levels of self-esteem, peer relations, social acceptance, academic achievement, and academic aspirations than peers with lower levels of life satisfaction. However, the majority of studies used life satisfaction as an outcome and a longitudinal study [80] indicated that class climate affects school-aged children’s life satisfaction more likely.

Further, as previous studies considered different measures of subjective wellbeing [5], only few studies used life satisfaction as a single indicator in association with either school-aged children's individual perceptions of class climate or the overall learning environment at the class-level. In general, many studies used a multi-dimensional instrument of school-aged children's wellbeing (for example [81, 82] or used the cognitive component of wellbeing (i.e. life satisfaction) as a single outcome measure [5, 32]. Thus, it is hardly possible to disentangle whether our results would have differed when using a multi-dimensional construct of wellbeing. Future research is therefore warranted in order to validate our findings with other indicators of wellbeing. However, many studies in research on child and adolescent wellbeing used school-aged children's general life satisfaction as a single indicator of mental and psychological wellbeing, which is increasingly acknowledged as an important outcome measure [32]. In this context, indicators and index-variables that have been used to measure the class climate in this study also differ from indicators that have been used in other studies. Therefore, it is hardly possible to fully compare our results with prior research findings.

Regarding measures of class climate, this study relied on self-reported information from school-aged children as the NEPS, unfortunately, does not provide detailed information on the class climate from teachers. This makes it impossible to compare school-aged children’s assessment of class climate with teachers’ perceptions in order to provide reliable information. The overall learning environment in terms of class climate was measured in this study as aggregated mean-levels of those indicators gathered at the individual-level. In line with evidence from educational research, investigating so-called compositional measures when school-aged children are organized into classes on the basis of for example ability or ethnicity (for example [41, 45, 47], we also used individual information of perceived class climate in this study by aggregating those measures as mean-levels to the class-level [50]. However, there are of course strengths and weaknesses in relation to research on the impact of compositional measures in classes or schools [47] that have been aggregated from information at the individual-level. However, in line with other studies examining compositional characteristics, we also controlled in this study for the individual-level variable when introducing the aggregated mean-level of class climate indicators at the class-level into the model. Thus, the remaining effect observed is therefore not confounded by the individual school-aged children's perceptions of class climate [50, 52]. Further, composition in class is often correlated with an array of school characteristics, from resources to the nature of peer relations to the quality of teachers [47]; making it hardly possible to identify the causal relationship between those compositional measures and young people’s outcomes [47, 50]. Information from parents or teachers on class climate dimensions could serve as a complement to children's reports in future studies.

Further, as the sample that has been used in this study contains school-aged children in grade 6 of lower secondary schools, we cannot clearly preclude that being in a new school and learning environment when having completed the transition from primary to lower secondary schools, accompanied by new classmates, new composition in class and new teachers, may affect school-aged children life satisfaction in both positive and negative ways. However, as school-aged children in our sample experienced the transition after grade 4, they have already completed more than one school year in this new learning environment when the survey took place. Thus, they are likely to be well-accustomed to their classmates, class composition, teachers and their teaching organization and practices. Longitudinal data would help to unravel these associations. Unfortunately, the data we used in this study stems from a longitudinal survey (NEPS), but we only can make use of cross-sectional data from one NEPS-wave (Starting Cohort 3, wave 2) because NEPS contains such a variety of indicators on class climate only in wave 2. Future longitudinal studies are therefore warranted in order to unravel the association between school transition, features of the learning environment and life satisfaction.

Unfortunately, as indicators of class climate in this study only refer to the teachers in German language class, general conclusions to the overall class climate cannot be drawn without caution. However, German language classes are instructions which are compulsory for school-aged children, particularly in lower secondary education in the German schooling system. Therefore, it is a school subject that takes place in the entire class with all grade 6 school-aged children which makes it plausible to refer to the German language teacher in order to measure the class climate from school-aged children's perspective. In how far, the perception of class climate indicators in German language classes are transferable to the overall class climate in class, is not possible with those measures on class climate which have been used in this study. Unfortunately, NEPS does not provide other, more general indicators of class climate. Therefore, general conclusions are, however, not possible.

Further, some methodological issues need to be addressed. Variations in life satisfaction among classes were low (ICC_classes_: 1.7% in the empty model). Studies, which investigated those school- or class-level characteristics on school-aged children's outcomes, concluded that the impact of those aggregate indicators at the class- or school-level over and above the contribution of the individual indicator on young people's wellbeing is rather small. As in prior studies only a small proportion of about 0.5% to 5% of the variation in outcomes, such as health or wellbeing, can be explained by school- or class-level features [17, 49, 54, 73, 83]. For instance, studies on school-aged children’s health behaviors reported an ICC of 7%-12% [53], whereas studies on school-aged children’s wellbeing generally reported much lower ICCs between schools and classrooms [4, 17, 54]. However, although life satisfaction as a cognitive dimension of subjective wellbeing [5] is mainly explained by individual characteristics or perceptions, researchers already urged to understand satisfaction with life as embedded in the ecological context of life, such as the interpersonal, social-familial, institutional and also the school context [4]. Finally, multicollinearity has to be considered, facing correlated class climate indicators, which are entered twice into the models–at individual as well as at school-level. In sensitivity analyses, indicators of class climate at individual as well as at class-level were introduced step-wise. Therein, coefficients showed very small changes in magnitude and direction, comparable to the changes in coefficients of class climate indicators between Model 1 and Model 3. This is an indicator for negligible bias in the coefficients due to multicollinearity.

## Conclusions

The perception of the school environment is of importance for young people’s wellbeing. This study investigated whether not only the individually perceived class climate is related to school-aged children’s life satisfaction, but whether the overall learning environment in school classes can also contribute to young people's life satisfaction. According to our results, the individual perception of class climate in different areas is closely linked to school-aged children’s life satisfaction, whereas the overall learning environment in classes showed only a partial and weak association with life satisfaction. Thus, the individually perceived class climate is important for the cognitive wellbeing of young people, not only because life satisfaction is an important facet of young people's overall wellbeing, but also because life satisfaction is closely linked to educational aspirations and educational engagement. Lastly, the fact that school is compulsory at least up to the tenth grade makes it even more important to ensure that the perceived class climate serves to enhance school-aged children’s wellbeing [17, 55].
